# Non-Target Effects of Beta-Cypermethrin on *Baryscapus dioryctriae* and Ecological Risk Assessment

**DOI:** 10.3390/insects16090948

**Published:** 2025-09-10

**Authors:** Jing Li, Tongtong Zuo, Sicheng Fei, Yuequ Chen, Xiangyu Zhang, Qi Chen, Liwen Song, Kaipeng Zhang

**Affiliations:** 1 Research Institute of Forest Protection, Jilin Provincial Academy of Forestry Sciences, Changchun 130033, China; lij9030@163.com (J.L.); ttz1983@126.com (T.Z.); fungi3@126.com (Y.C.); 18844440155@163.com (X.Z.); 2 Jilin Province Main Forest Diseases and Insect Pests Monitoring, Epidemic Prevention Technology Innovation Center, Changchun 130033, China; 3 Laboratory of Entomology, Plant Sciences Group, Wageningen University & Research, 6700 AA Wageningen, The Netherlands; sicheng.fei@wur.nl; 4 Jilin Provincial Key Laboratory of Animal Resource Conservation and Utilization, School of Life Sciences, Northeast Normal University, Changchun 130024, China; chenq766@nenu.edu.cn; 5 Key Laboratory of Vegetation Ecology, Ministry of Education, Northeast Normal University, Changchun 130024, China; 6 Jilin Provincial Engineering Laboratory of Avian Ecology and Conservation Genetics, Northeast Normal University, Changchun 130024, China; 7 Jilin Provincial International Cooperation Key Laboratory for Biological Control of Agricultural Pests, Changchun 130024, China

**Keywords:** *Baryscapus dioryctriae*, beta-cypermethrin, biological control, detoxification enzymes, ecological risk, integrated pest management, oxidative stress, sublethal effects

## Abstract

Beta-cypermethrin is widely applied in Korean pine (*Pinus koraiensis* Siebold & Zucc.) seed orchards to control cone- and seed-infesting moths (e.g., *Dioryctria* spp.), yet its Wsublethal risks to non-target beneficial arthropods remain insufficiently characterized. Here, we systematically evaluated the ecological and physiological consequences of beta-cypermethrin exposure on the key parasitoid wasp *Baryscapus dioryctriae* Yang & Song, an important biological control agent in *P. koraiensis* forests. Adult wasps were exposed to LC_30_ and LC_50_ residue concentrations, and sublethal effects were quantified across reproductive, developmental, and biochemical endpoints over two generations. Sublethal exposure resulted in significant reductions in parasitism rates and offspring emergence, as well as altered developmental durations and adult longevity in both F_0_ and F_1_ generations. Enzymatic assays revealed time-dependent activation of detoxification enzymes (GST, CarE, AChE) alongside suppression of antioxidant defenses (CAT strongly; SOD early with partial recovery; POD biphasic), consistent with a sustained oxidative-stress burden. LC-MS/MS residue analysis further confirmed the accumulation and slow clearance of both beta-cypermethrin and its metabolite 3-phenoxybenzoic acid (PBA) within parasitoid tissues. These findings collectively demonstrate that even non-lethal concentrations of beta-cypermethrin can undermine the ecological fitness and persistence of *B. dioryctriae*, posing a tangible threat to the sustainability of biological control services. To safeguard beneficial parasitoids, integrated pest management strategies must incorporate selective insecticide use and exposure mitigation, especially in forest habitats where biological control is indispensable.

## Simple Summary

Foresters often use the insecticide beta-cypermethrin to protect Korean pine seed orchards from cone- and seed-feeding moths. However, such sprays can also affect helpful insects that naturally control these pests. We studied a tiny wasp, *Baryscapus dioryctriae*, which parasitizes the moths and is important for biological control. Adult wasps were exposed to light residues of beta-cypermethrin similar to what they could contact on treated surfaces. Even at non-lethal levels, exposed wasps parasitized fewer hosts and produced fewer offspring. Their development slowed, adults lived for a shorter time, and their normal detoxification and stress-defense systems were disturbed. Chemical analysis showed that beta-cypermethrin and one of its breakdown products remained in wasp bodies for several days, indicating slow clearance. These results show that routine spraying can quietly weaken beneficial wasps and their next generation, reducing natural pest control. To support healthy forests, pest management should favor selective products, reduce exposure of natural enemies, and integrate spraying with biological control.

## 1. Introduction

Parasitoid wasps play a pivotal role in the suppression of agricultural and forest pests and are integral to sustainable pest management systems worldwide [1,2,3]. Their ability to regulate pest populations reduces reliance on synthetic insecticides, supporting ecological balance and long-term crop productivity [4,5,6]. However, the extensive use of broad-spectrum insecticides, particularly pyrethroids such as beta-cypermethrin, poses serious risks to non-target beneficial insects including parasitoids and pollinators [7,8,9,10]. In our study region, beta-cypermethrin is operationally deployed against cone- and seed-infesting *Dioryctria* spp. in *Pinus koraiensis* Siebold & Zucc. seed orchards, motivating an assessment of its compatibility with the resident parasitoid *Baryscapus dioryctriae* Yang & Song.

Pyrethroids act mainly on voltage-gated sodium channels in insect nervous systems, resulting in rapid paralysis and mortality of target pests [10,11]. Their high efficacy, low mammalian toxicity, and environmental persistence have led to widespread adoption in forestry and agriculture [4,12]. However, these properties also increase their hazard to non-target arthropods, which may experience both acute and chronic effects [8,13,14]. Intensive and repeated applications disrupt natural enemy populations, weakening biological control and often resulting in pest resurgence or secondary outbreaks [4,15,16].

Emerging evidence highlights that the impact of insecticides extends far beyond immediate mortality, with sublethal and transgenerational effects increasingly recognized as significant threats to ecosystem services [8,15,17,18,19]. Sublethal exposure can impair parasitoid behavior, such as host-seeking, oviposition, and locomotion, leading to reduced parasitism rates and population growth [8,17,20,21]. Transgenerational consequences—including decreased offspring fitness, survival, and altered development—have been reported in both parasitoids and predators [14,18,19].

A key mechanistic basis for these adverse effects is oxidative stress, a process by which insecticides disrupt redox homeostasis and generate excessive reactive oxygen species (ROS), leading to cellular damage [7,11,12,22,23]. Beneficial insects, including parasitoids, rely on a network of antioxidant enzymes such as superoxide dismutase (SOD), catalase (CAT), glutathione S-transferase (GST), and carboxylesterase (CarE) to mitigate oxidative damage [24,25,26,27]. Sublethal insecticide exposure can inhibit or dysregulate these defenses, increasing susceptibility to further stressors [20,28,29]. Recent studies have also revealed that insecticide metabolites, such as 3-phenoxybenzoic acid (3-PBA), may accumulate in non-target organisms and contribute to persistent physiological disruption [12,30].

While the non-target risks of insecticides for pollinators and some predators have been extensively studied [9,13,31,32,33], far less attention has been paid to forest parasitoids—especially newly described species with high biocontrol value [2,3,34,35]. For instance, the pupal parasitoid *B. dioryctriae* has demonstrated considerable potential in suppressing cone and seed pests in pine forests [3,34,35], but the environmental compatibility of pyrethroid-based pest management in these systems remains largely unassessed.

Given these knowledge gaps, this study examines the sublethal and transgenerational effects of beta-cypermethrin exposure on the key life history traits, antioxidant enzyme activities, and detoxification metabolism of *B. dioryctriae* across two generations. We also quantify the internal residues of beta-cypermethrin and its principal metabolite 3-PBA in parasitoid tissues to evaluate detoxification and clearance capacity. By integrating behavioral, physiological, and toxicological endpoints, our research provides a comprehensive risk assessment for non-target effects of pyrethroids on parasitoids and offers critical insights for the development of more selective and sustainable integrated pest management strategies [4,10,16].

## 2. Materials and Methods

### 2.1. Insect Rearing

Adult *B. dioryctriae* were obtained from naturally parasitized *Dioryctria pryeri* Ragonot pupae collected at a Korean pine (*P. koraiensis*) seed orchard managed by the Lushuihe Forestry Bureau (Baishan City, China). The parasitoids were reared in the laboratory on *Galleria mellonella* (Lepidoptera: Pyralidae) pupae, which were supplied by a breeding colony at the Tianjin Lepidoptera Research Center. *G. mellonella* larvae were maintained on an artificial diet until pupation. For parasitization assays, individual *G. mellonella* pupae were placed singly in 15 mm × 65 mm glass tubes. All insects were kept in climate-controlled chambers at 25 ± 1 °C, 50 ± 1% relative humidity (RH), and a 16:8 h light:dark photoperiod. Only 1-day-old adult female *B. dioryctriae* were used in all experiments. Here we used the standardized factitious host *G. mellonella* to ensure continuous host supply and to reduce variability in host size, nutritional status, and cuticular properties across replicates.

### 2.2. Insecticide Preparation and Residue Film Method

Technical beta-cypermethrin (98% purity; ZZstandard, Shanghai, China) was used to prepare stock solutions. The insecticide was first dissolved in acetone and then diluted with distilled water containing 0.1% (*v*/*v*) Triton X-100 to obtain a series of test concentrations. Five concentrations (0.2, 1, 5, 25, and 125 mg/L) were prepared by serial dilution and used to determine concentration–response curves, from which LC_30_ and LC_50_ values were estimated. These five concentrations were selected after a preliminary range-finding assay (n = 3) to bracket ~10–90% mortality at 24 h, ensuring robust Probit estimation. A control solution of 0.1% Triton X-100 in water was prepared in parallel. For the residue film bioassay, 0.5 mL of each insecticide or control solution was pipetted into a clean glass tube (10 cm length × 3 cm diameter) and the tube was slowly rotated to coat the entire inner surface. The solvent was allowed to evaporate by air-drying, leaving a thin insecticide residue film on the tube walls. To facilitate comparison with field deposits, we also expressed residue-film exposure as a surface load (ng/cm^2^) calculated from the applied concentration, coating volume (0.5 mL = 0.0005 L per tube), and the inner lateral surface area (A ≈ 94.25 cm^2^; A = 2πrh with r = 1.5 cm and h = 10 cm). Under this geometry, LC_30_ (1.359 mg/L) corresponds to ~7.21 ng/cm^2^, and LC_50_ (8.027 mg/L) to ~42.58 ng/cm^2^, assuming uniform film distribution. Reporting residue as a surface load (ng/cm^2^) in glass-tube residual-film assays follows established parasitoid ecotoxicology protocols using acetone coating and solvent evaporation [36,37].

### 2.3. Acute Toxicity Assay

Groups of twenty newly emerged female wasps (<24 h old) were introduced into glass tubes treated with insecticide residue films and allowed to contact the surface for 1 h. Following exposure, wasps were transferred to clean tubes and maintained with 10% (*w*/*v*) honey solution provided on cotton balls. Mortality was assessed after 24 h; individuals were considered dead if they failed to respond to gentle probing with a fine brush. Each treatment, including the control, was replicated ten times. The assay was considered valid if control mortality was below 10%. Lethal concentrations (LC_30_ and LC_50_) at 24 h were estimated by probit analysis using PoloPlus 1.0 software (LeOra Software, Petaluma, CA, USA).

### 2.4. Reproductive Performance, Life History Parameters and Developmental Durations

The effects of insecticide exposure on parasitism and developmental parameters were evaluated for both the parental (F_0_) and offspring (F_1_) generations. For each treatment group, batches of F_0_ females (previously exposed to LC_30_ or LC_50_) were allowed to parasitize *G. mellonella* pupae at a ratio of 1:4 (host:female) in glass tubes containing a honey solution for feeding. After oviposition, the pupae were maintained under rearing conditions. The number of parasitized hosts (mummies), offspring per female (total emerged adults per female), emergence rate (% of adults emerged from mummies), and sex ratio (female:male) were recorded for the F_0_ generation.

Developmental durations (egg to adult) and adult longevity of emerged offspring were also monitored. Developmental durations were divided into the following stages: (1) egg–larva: from parasitization (exposure to host) until the appearance of visible mummification (darkened and hardened host shell); (2) pupa–adult: from mummification to adult emergence (exit from host puparium); (3) adult longevity: from adult emergence to natural death under feeding conditions (10% honey solution). The F_1_ generation (offspring of F_0_ females) was reared under identical conditions, and a subset of F_1_ females was similarly tested to determine transgenerational effects on parasitism rate, fecundity, developmental duration, and longevity.

### 2.5. Enzyme Activity Assays

Enzyme activities were measured in adult female wasps exposed to the sublethal LC_30_ concentration. Surviving wasps were sampled at 24, 48, 72, 96, 120, 144, and 168 h post-exposure. At each time point, wasps were collected, flash-frozen in liquid nitrogen, and stored at −80 °C until analysis. Activities of glutathione S-transferase (GST), superoxide dismutase (SOD), catalase (CAT), peroxidase (POD), carboxylesterase (CarE) and acetylcholinesterase (AChE) were assayed using commercial enzyme assay kits (Suzhou Dream Biology, Suzhou, China) according to the manufacturers’ protocols. Enzyme activities were normalized to fresh weight (FW) of insect tissue. Results were expressed as: GST nmol/min/g FW, AChE nmol/min/g FW, CarE U/g FW, CAT μmol/min/g FW, POD U/g FW and SOD U/g FW.

### 2.6. LC-MS/MS Analysis of Residues and Metabolites

Beta-cypermethrin and its primary metabolite 3-phenoxybenzoic acid (PBA) were quantified in adult parasitoids at multiple time points (24, 48, 72, 96, 120, 144, and 168 h) post-LC_30_ exposure. To investigate detoxification dynamics, live and dead individuals were analyzed separately at each interval. Insects were rinsed sequentially with deionized water and 75% ethanol to remove external residues, then flash-frozen in liquid nitrogen. Each sample (n = 3 replicates per group) was homogenized in ice-cold 50% methanol, sonicated for 15 min, and centrifuged at 12,000× *g* (4 °C) for 10 min. The supernatant was mixed 1:1 (*v*/*v*) with methanol:acetonitrile (1:1) and re-centrifuged to remove precipitates.

Chromatographic separation was performed on an Agilent Eclipse Plus C18 column (2.1 × 100 mm, 1.8 μm) maintained at 40 °C. The mobile phases consisted of solvent A (0.1% formic acid in water) and solvent B (acetonitrile), with a linear gradient from 20% to 80% B over 10 min at a flow rate of 0.3 mL/min. Detection was achieved using an Agilent 6460 triple quadrupole LC-MS/MS equipped with electrospray ionization. Beta-cypermethrin was analyzed in positive ion mode (*m*/*z* 415.1→163.1), and PBA in negative ion mode (*m*/*z* 213.0→169.0). Quantification was based on external calibration curves (*R*^2^ > 0.99) and deuterated internal standards. Residue amounts were normalized to sample fresh weight and reported as ng/g FW.

### 2.7. Statistical Analysis

All statistical analyses were performed using SPSS v27.0 (IBM Corp., Armonk, NY, USA), GraphPad Prism 8.0 (GraphPad Software, San Diego, CA, USA) and PoloPlus (LeOra Software, Petaluma, CA, USA). Mortality data were analyzed by probit regression (PoloPlus) to estimate LC_30_ and LC_50_ values with 95% confidence intervals. Reproductive parameters and enzyme activities were compared among groups using one-way ANOVA followed by Tukey’s HSD test. Two-way ANOVA was employed to evaluate the main effects and interactions for developmental durations [Generation (F_0_, F_1_) × Treatment (CK, LC_30_, LC_50_)] and for enzyme activities [Treatment (control, LC_30_) × Time (24–168 h)]. When significant interactions (*p* < 0.05) were detected, further analyses were performed: egg–larva duration (significant Generation × Treatment interaction, *p* = 0.001) was examined using within-generation pairwise Tukey HSD tests and an a priori cross-generational comparison (F_0_-LC_50_ vs. F_1_-LC_50_); pupa–adult duration (significant Treatment effect, *p* < 0.001) was analyzed by generation-specific Tukey HSD tests. Time-course enzyme activity data were additionally analyzed by independent samples *t*-tests (with Welch’s correction if variances were unequal, as determined by Levene’s test) at each time point. For residue dynamics (beta-cypermethrin/PBA), two-way ANOVA [Time × Vital Status (Live/Dead)] was used, followed by pairwise *t*-tests (Live vs. Dead) at each time point with Bonferroni adjustment. All data satisfied the assumptions of ANOVA (normality: Shapiro–Wilk test; homogeneity of variance: Levene’s test). Data are presented as means ± standard error (SE). Statistical significance threshold was set at α = 0.05.

## 3. Results

### 3.1. Acute Toxicity of Beta-Cypermethrin to B. Dioryctriae

The acute contact bioassay yielded an LC_30_ of 1.359 mg/L (95% CI 0.276–3.476 mg/L) and an LC_50_ of 8.027 mg/L (95% CI 3.098–23.794 mg/L) at 24 h post-exposure. The fitted probit model showed a slope of 0.68 ± 0.148 and an *R*^2^ of 0.98 (Table S1), indicating moderate tolerance at sublethal doses. Based on these results, LC_30_ and LC_50_ were selected to represent sublethal and high exposure levels for subsequent experiments.

### 3.2. Reproductive Performance

**Figure 1:**
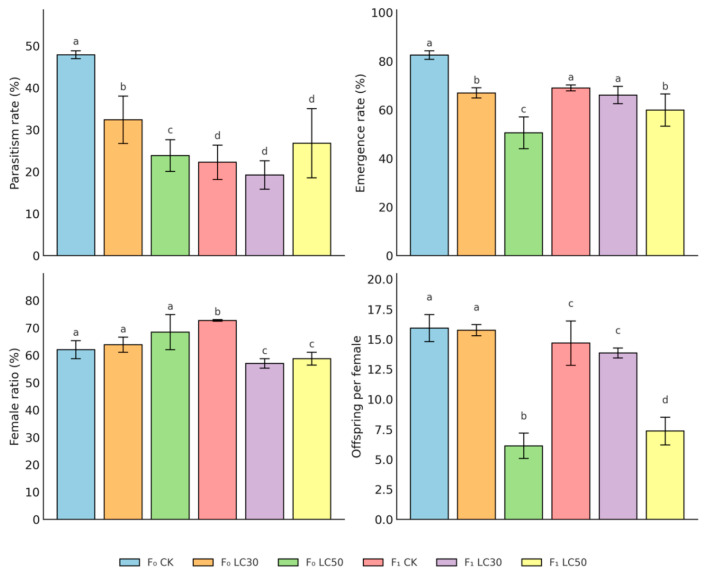
Reproductive performance of *B. dioryctriae* under sublethal beta-cypermethrin exposure. Each panel shows one reproductive indicator (parasitism rate, emergence rate, female proportion, and offspring per female) across two generations (F_0_ and F_1_) under control (CK), LC_30_ and LC_50_ conditions. Bars represent mean ± SE (n = 3). Different letters above bars indicate significant differences between groups (Tukey’s HSD test, *p* < 0.05).

High beta-cypermethrin exposure (LC_50_) severely compromised reproductive success in the parental (F_0_) generation of *B. dioryctriae* (Figure 1; Table 1). Compared to controls (F_0_ CK), LC_50_ exposure reduced parasitism rates by 50% (23.84% vs. 47.85%, *p* = 0.0022), emergence rates by 39% (50.50% vs. 82.48%, *p* = 0.0003), and offspring production per female by 62% (6.12 vs. 15.92, *p* < 0.0001) (Table S2). In contrast, sublethal exposure (LC_30_) showed no significant effects on these parameters in F_0_ (all *p* > 0.05), though a numerical reduction in parasitism (32.37% vs. 47.85%) was observed. Notably, female ratio remained unaffected across all F_0_ treatments (*p* > 0.05).

In the F_1_ generation, high exposure (LC_50_) significantly reduced offspring production by 50% compared to controls (7.35 vs. 14.67, *p* = 0.0005), while sublethal exposure (LC_30_) had no significant impact (13.85 vs. 14.67, *p* > 0.05) (Table 1 and Table S2). Two-way ANOVA revealed significant treatment effects on parasitism (*p* = 0.0042) and emergence rates (*p* = 0.0039) in F_1_ (Table S3). However, post hoc tests showed no pairwise differences between specific treatment groups (CK vs. LC30, CK vs. LC50, LC30 vs. LC50) for these parameters (all *p* > 0.05). Female ratio showed no significant variation among F_1_ treatments (*p* > 0.05).

A significant treatment × generation interaction was detected for offspring production (*p* = 0.0003, Table S3), with LC_50_ exposure causing more severe reductions in F_0_ (−62%) than in F_1_ (−50%). This generational attenuation suggests partial recovery in offspring production capacity, though LC_50_ effects remained substantial in both generations.

Overall, high insecticide exposure (LC_50_) consistently impaired reproductive performance across generations, particularly in reducing offspring production. Sublethal exposure (LC_30_) showed minimal statistically detectable effects, highlighting the critical concentration-dependent nature of these impacts.

### 3.3. Developmental Durations

Sublethal beta-cypermethrin exposure significantly altered developmental timing in *B. dioryctriae* across generations (Table 2; Figure S1). In the F_0_ generation, egg–larva duration was numerically shorter under LC_30_ (12.33 ± 0.33 days) than CK (13.67 ± 0.33 days), but not statistically significant (Tukey HSD: *p* = 0.353; Table S4). Pupa–adult duration was significantly prolonged in LC_50_ (5.67 ± 0.33 days) versus CK (4.00 ± 0.00 days; *p* = 0.002) and LC_30_ (4.33 ± 0.33 days; *p* = 0.010). Adult longevity showed no significant differences (11.12 ± 0.73 vs. 10.25 ± 0.40 days).

In the F_1_ generation, egg–larva duration was significantly shorter in LC_50_ (11.67 ± 0.67 days) versus CK (14.33 ± 0.67 days; *p* = 0.013) and LC_30_ (14.33 ± 0.33 days; *p* = 0.013). Pupa–adult duration was prolonged in LC_50_ (5.67 ± 0.33 days) versus CK (3.67 ± 0.33 days; *p* < 0.001) and LC_30_ (4.00 ± 0.00 days; *p* < 0.001). Adult longevity in LC_50_ (13.65 ± 0.95 days) was numerically longer but not statistically significant.

A significant transgenerational effect was observed in egg–larva duration, with the F_1_-LC_50_ group developing faster than the F_0_-LC_50_ group (*p* = 0.030). No significant transgenerational differences were detected for pupa–adult duration.

These results demonstrate that beta-cypermethrin accelerated egg–larval development, specifically in F_1_-LC_50_, consistently delayed pupa–adult development at LC_50_ in both generations, and exerted minimal effects under LC_30_ and on adult longevity.

### 3.4. Enzyme Activity

**Figure 2:**
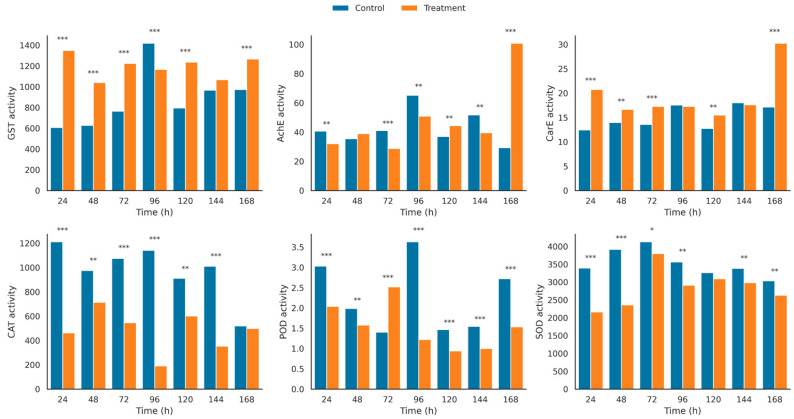
Bar plots showing the activities of six key enzymes (glutathione S-transferase (GST), acetylcholinesterase (AChE), carboxylesterase (CarE), catalase (CAT), peroxidase (POD), and superoxide dismutase (SOD) in *B. dioryctriae* adults after beta-cypermethrin (LC_30_) exposure. Enzyme activities were measured at seven time points (24 to 168 h). Bars represent the mean ± standard error (n = 3). Asterisks indicate statistically significant differences between treatment and control groups at each time point (*, *p* < 0.05; **, *p* < 0.01; ***, *p* < 0.001; independent *t*-test).

Sublethal beta-cypermethrin (LC_30_) exposure induced time-dependent alterations in the enzymatic profile of *B. dioryctriae* adults (Figure 2 and Figures S2; Table S5). Detoxification enzymes exhibited pronounced activation: GST activity was consistently elevated across all time points (*p* < 0.0001), peaking at 24 h (1349.56 ± 7.45 nmol/min/g FW) and 168 h (1267.25 ± 19.90 nmol/min/g FW), representing a 122% and 30% increase over controls, respectively. CarE activity increased significantly (*p* < 0.0001), with maximal induction at 24 h (20.75 ± 0.15 vs. 12.46 ± 0.25 U/g FW) and 168 h (30.26 ± 1.33 vs. 17.13 ± 0.25 U/g FW). AChE showed dynamic fluctuations: transient suppression at 24–72 h (e.g., 32.12 ± 0.30 vs. 40.70 ± 1.50 nmol/min/g FW at 24 h), followed by a 244% surge at 168 h (100.88 ± 1.66 vs. 29.32 ± 0.84 nmol/min/g FW).

Antioxidant defenses were significantly suppressed: CAT activity was inhibited by 62–83% throughout the exposure period (*p* < 0.0001), most severely at 96 h (194.57 ± 4.89 vs. 1142.06 ± 49.05 μmol/min/g FW). SOD activity decreased by 36–40% during 24–48 h (e.g., 2163.96 ± 18.43 vs. 3380.67 ± 32.15 U/g FW at 24 h, *p* < 0.0001), with partial recovery at later stages. POD activity displayed biphasic regulation: initial inhibition (e.g., 2.04 ± 0.03 vs. 3.30 ± 0.08 U/g FW at 24 h), but elevated at 72 h (2.52 ± 0.06 vs. 1.40 ± 0.03 U/g FW).

Two-way ANOVA (Table S6) confirmed statistically significant effects of treatment (*p* < 0.0001), time (*p* < 0.0001), and their interaction (*p* < 0.0001) for all six enzymes. This coordinated response indicates that LC_30_ exposure simultaneously activates detoxification pathways and overwhelms antioxidant capacity, reflecting a sustained oxidative stress state.

### 3.5. Residue Dynamics

**Figure 3:**
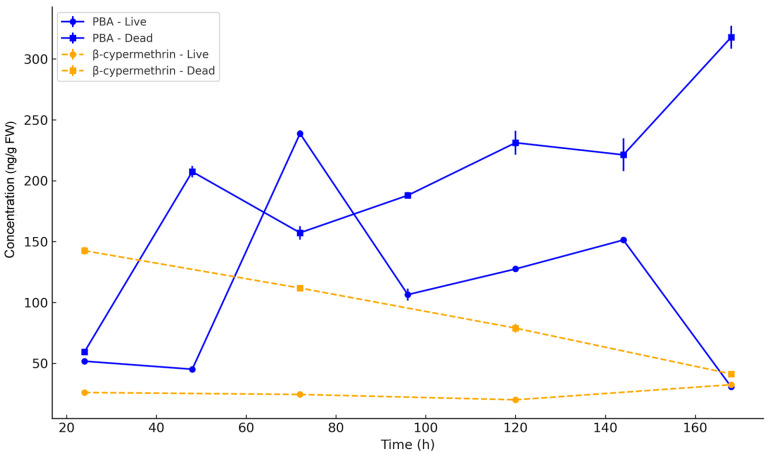
Accumulation of beta-cypermethrin and its primary metabolite 3-phenoxybenzoic acid (PBA) in *B. dioryctriae* adults after sublethal (LC_30_) exposure. Residue concentrations (ng/g FW) were measured in live and dead wasps at seven time points. Data are presented as mean ± SE (n = 3).

To evaluate internal pesticide burden and detoxification, the levels of beta-cypermethrin and its primary metabolite PBA were tracked in *B. dioryctriae* adults over 168 h post-LC_30_ exposure (Figure 3; Table S7). In live parasitoids, beta-cypermethrin remained consistently low (24 h: 26.01 ± 0.53 ng/g; 168 h: 32.43 ± 0.63 ng/g), while dead individuals showed significantly higher residues that decreased sharply from 142.54 ± 3.04 ng/g (24 h) to 41.26 ± 0.44 ng/g (168 h) (*p* < 0.001 at all time points; Table S8).

PBA dynamics revealed an inverse pattern: Live wasps exhibited transient accumulation (peak: 238.72 ± 2.78 ng/g at 72 h) followed by clearance to 30.62 ± 0.43 ng/g (168 h). Dead wasps showed progressive accumulation from 59.36 ± 1.58 ng/g (24 h) to 317.86 ± 9.44 ng/g (168 h).

Two-way ANOVA confirmed strong effects of: Time (*p* < 0.0001), Vital status (*p* < 0.0001), and Time × Status interaction (beta-cypermethrin: *p* = 0.0158; PBA: *p* = 0.0022; Table S8).

These patterns demonstrate active biotransformation of beta-cypermethrin to PBA in live parasitoids, whereas dead individuals accumulated PBA due to impaired clearance. Sublethal exposure thus triggers dynamic detoxification processes directly linked to survival outcomes.

## 4. Discussion

Our findings provide robust evidence that sublethal exposure to beta-cypermethrin can significantly impair the reproductive performance, development, and physiological homeostasis of *B. dioryctriae*, with some effects persisting into the subsequent generation. These results are consistent with an expanding body of literature demonstrating that insecticides, even at non-lethal concentrations, can undermine natural enemy efficacy and threaten the sustainability of biological control [8,13,19].

### 4.1. Reproductive Suppression and Developmental Disruption

Exposure to sublethal (LC_30_ and LC_50_) concentrations of beta-cypermethrin led to a pronounced decline in parasitism rates, fecundity, and offspring emergence in *B. dioryctriae*. Similar findings have been reported in other parasitoid systems, where sublethal pyrethroid exposure reduced reproductive output, host-finding ability, and longevity [17,19,20,21]. The observed reduction in offspring number and eclosion aligns with the notion that sublethal stressors can induce hidden demographic costs, thereby lowering population growth rates and diminishing long-term biocontrol capacity [14,38]. Notably, our results did not reveal a significant shift in sex ratio, contrasting with some reports of male-biased offspring under stress [18], suggesting species-or context-specific reproductive allocation responses.

### 4.2. Transgenerational Effects

The persistent reduction in F_1_ offspring performance, even when these individuals were not directly exposed to beta-cypermethrin, highlights the need to consider multigenerational impacts in risk assessments [18,19]. Plausible mechanisms include maternal effects, disruption of symbiotic microbiota, or epigenetic modifications [15,39]. However, these mechanisms were not directly assayed in this study: we did not quantify maternal provisioning, characterize microbial communities, or evaluate epigenetic marks; therefore, they should be regarded as hypotheses consistent with published evidence and testable directions for future work (e.g., microbiome profiling with perturbation, cross-fostering, RNA-seq/methylome assays). For example, in aphid parasitoids, pyrethroid-exposed mothers produced less fit progeny with reduced developmental rates and lower reproductive potential [17].

Because all assays were performed on the factitious host *G. mellonella*, host-related traits (e.g., size, hemolymph composition, cuticle thickness) could modulate parasitoid performance and potentially interact with sublethal insecticide stress. We therefore regard our laboratory results as a standardized benchmark rather than an exhaustive depiction of field complexity. To confirm operational compatibility with biological control in Korean pine seed orchards, future semi-field/field trials on the target *Dioryctria* spp. are warranted.

### 4.3. Enzyme Dynamics and Mechanistic Interpretation

Under LC_30_ exposure, enzyme activities followed a time-structured modulation rather than a uniform shift: GST and CarE were induced across most sampling points, AChE showed a transient decrease (24–72 h) with a late rebound (168 h), and antioxidant capacity was concurrently constrained—CAT remained depressed, SOD was suppressed early with partial recovery, and POD was biphasic. These trajectories indicate a redistribution of metabolic resources toward biotransformation/detoxification under sustained oxidative challenge while redox buffering is limited, a pattern congruent with established views of pyrethroid-driven oxidative stress, compensatory regulation, and hormesis-like responses [14,15,24,26,40]. Such compensation is energetically costly and plausibly contributes to the observed reductions in parasitism/offspring emergence and shortened adult longevity, i.e., fitness trade-offs under persistent stress [14,15,21]. The persistence of beta-cypermethrin and PBA residues over seven days provides a mechanistic basis for prolonged compensatory demand and late-stage AChE/POD adjustments [26,30,40].

### 4.4. Residue Accumulation and Metabolite Risks

LC-MS/MS analyses confirmed that both beta-cypermethrin and its major metabolite 3-PBA persist in parasitoid tissues for several days post-exposure. While carboxylesterases facilitate partial detoxification, the slow clearance rates and persistence of 3-PBA raise concerns for chronic toxicity [12,14,30]. Notably, 3-PBA has been shown to exert neurotoxic effects in vertebrates and may similarly contribute to long-term impairment in insects [30]. The extended presence of these residues implies that parasitoids remain at risk even after environmental concentrations have declined, increasing the likelihood of cumulative and delayed sublethal effects.

### 4.5. Ecological and IPM Implications

The cumulative impact of reduced reproductive success, impaired physiological function, and residue accumulation in parasitoid populations has important ecological ramifications. Sustained suppression of natural enemy abundance or performance can destabilize pest–natural enemy dynamics, leading to pest resurgence and undermining integrated pest management [4,10,16]. This risk is especially salient in forest systems, where natural enemies such as *B. dioryctriae* are essential for regulating pest outbreaks and maintaining tree health [16,19,34,35].

Our findings thus underscore the urgent need to minimize reliance on broad-spectrum insecticides and to integrate selective chemistries, biological control, and habitat management for effective and sustainable pest suppression [1,4,16]. Adoption of selective application timing, reduced-risk compounds, and conservation strategies (such as providing floral resources or refugia) can help safeguard parasitoid populations and their ecosystem services [5,6,35].

### 4.6. Future Directions

Further research should test the population- and community-level consequences of sublethal and transgenerational insecticide effects under semi-field and field conditions, ideally within target *Dioryctria* systems in Korean pine seed orchards. To improve lab–field inference, future studies should pair standardized residue metrics (e.g., surface load) with measured field deposits and explicitly evaluate how host identity modulates parasitoid responses and interacts with sublethal stress. Mechanistic hypotheses proposed here—maternal effects, microbiota disruption, and epigenetic regulation—remain untested in our system and warrant dedicated manipulative assays (e.g., microbiome perturbation and cross-fostering) alongside omics approaches to resolve causality [3,19,39]. Advances in genomics and transcriptomics offer new opportunities to identify genetic markers of detoxification capacity (e.g., P450s, GSTs, CarEs) and to develop resistant or robust natural enemy strains [3,40].

In conclusion, our findings reveal complex and enduring impacts of beta-cypermethrin exposure on a key parasitoid species, reinforcing the necessity for ecologically informed pesticide risk assessments and a shift toward more sustainable, parasitoid-compatible pest management paradigms.

## Figures and Tables

**Table 1:** Reproductive performance of *B. dioryctriae* under beta-cypermethrin exposure.

Group	Parasitism Rate (%)	Emergence Rate (%)	Female Ratio (%)	Offspring per Female
F_0_ CK	47.85 ± 0.93 a	82.48 ± 1.74 a	62.03 ± 3.28 a	15.92 ± 1.13 a
F_0_ LC_30_	32.37 ± 5.62 b	66.94 ± 2.09 b	63.83 ± 2.75 a	15.75 ± 0.46 a
F_0_ LC_50_	23.84 ± 3.78 c	50.50 ± 6.52 c	68.41 ± 6.43 a	6.12 ± 1.05 b
F_1_ CK	22.26 ± 4.09 d	68.98 ± 1.19 a	72.66 ± 0.30 b	14.67 ± 1.85 c
F_1_ LC_30_	19.21 ± 3.39 d	66.06 ± 3.53 a	56.95 ± 1.73 c	13.85 ± 0.42 c
F_1_ LC_50_	26.80 ± 8.25 d	59.85 ± 6.62 b	58.73 ± 2.36 c	7.35 ± 1.16 d

Note: Values are presented as mean ± SE (n = 3). Different letters indicate significant differences (Tukey’s HSD, *p* < 0.05). Offspring per female refers to the total number of emerged adults per female.

**Table 2:** Developmental duration and adult longevity (days) of *B. dioryctriae* under sublethal beta-cypermethrin exposure.

Generation	Treatment	Egg–Larva Duration (d)	Pupa–Adult Duration (d)	Adult Longevity (d)
F_0_	CK	13.67 ± 0.33 a	4.00 ± 0.00 a	10.25 ± 0.40
	LC_30_	12.33 ± 0.33 a	4.33 ± 0.33 a	11.12 ± 0.73
	LC_50_	14.00 ± 0.00 a	5.67 ± 0.33 b	10.60 ± 0.40
F_1_	CK	14.33 ± 0.67 b	3.67 ± 0.33 a	11.01 ± 0.65
	LC_30_	14.33 ± 0.33 b	4.00 ± 0.00 a	11.52 ± 0.63
	LC_50_	11.67 ± 0.67 a	5.67 ± 0.33 b	13.65 ± 0.95

Note: Values are presented as mean ± SE (n = 3 for all treatments). Different letters within columns indicate significant differences (Tukey HSD, α = 0.05). Two-way ANOVA reveals a significant interaction between generation and treatment for egg–larva duration (*p* = 0.001), a significant treatment effect on pupa–adult duration (*p* < 0.001), and no significant effects on adult longevity. Tukey’s HSD tests are provided in Table S4.

## Data Availability

Data supporting this study are available upon reasonable request.

## Supplementary Materials

The following supporting information can be downloaded at: https://www.mdpi.com/article/10.3390/insects16090948/s1, Table S1: Toxicity parameters of beta-cypermethrin on adult *Baryscapus dioryctriae* after 24 h exposure (n = 200); Table S2: Tukey HSD test results for reproductive performance in *Baryscapus dioryctriae*; Table S3: Two-way ANOVA summary for reproductive performance of *Baryscapus dioryctriae*; Table S4: Pairwise Tukey HSD test results for developmental durations of *Baryscapus dioryctriae*; Table S5: Enzyme activities in *Baryscapus dioryctriae* adults under beta-cypermethrin exposure; Table S6: Two-way ANOVA summary for enzyme activities of *Baryscapus dioryctriae*; Table S7: Levels of β-cypermethrin and its primary metabolite 3-phenoxybenzoic acid (PBA) in *Baryscapus dioryctriae* adults after LC_30_ exposure; Table S8: Summary of statistical test results (PBA vs. β-cypermethrin); Figure S1: Effects of sublethal β-cypermethrin exposure on the developmental durations and adult longevity of *Baryscapus dioryctriae*. Bar plots show the egg–larva duration, pupa–adult duration, and adult longevity across two generations (F_0_ and F_1_) under control (CK), LC_30_, and LC_50_ treatments. Data are presented as mean ± SE (n = 3 biological replicates). Significant differences between groups were determined using Tukey’s HSD test (*p* < 0.05), with asterisks and p-values indicated above the corresponding bars (see Table S4 for detailed statistics); Figure S2: Trends in the activities of six key enzymes in *Baryscapus dioryctriae* adults under beta-cypermethrin (LC_30_) exposure across seven time points (24–168 h). Each panel represents one enzyme: glutathione S-transferase (GST), acetylcholinesterase (AchE), carboxylesterase (CarE), catalase (CAT), peroxidase (POD), and superoxide dismutase (SOD). Data are presented as mean ± standard error (n = 3). Solid lines denote control and treatment groups. Significant interaction effects (treatment × time) were detected for all enzymes (Two-way ANOVA, *p* < 0.0001). Full enzyme activity values are provided in Table S5.
