# Supplementary material for: Non-Target Effects of Beta-Cypermethrin on Baryscapus dioryctriae and Ecological Risk Assessment
Source: Insects. 2025 Sep 10;16(9):948. doi: 10.3390/insects16090948 (PMC12471256; doi:10.3390/insects16090948)
Supplement: Supplementary file 1 [file insects-16-00948-s001.zip › insects-3822484-supplementary.pdf]

Non-target effects of beta-cypermethrin on *Baryscapus dioryctriae* and ecological risk assessment

Supplementary Information

Table S1. Toxicity parameters of beta-cypermethrin on adult *Baryscapus dioryctriae* after 24 h exposure (n=200).

Slope ± SE	LC_{30} (mg/L) (95% CI)	LC_{50} (mg/L) (95% CI)	R^2	Regression equation
0.68 ± 0.15	1.359 (0.276-3.476)	8.027 (3.098-23.794)	0.98	y=4.9x +5.6

Note: The slope refers to the probit regression model of mortality across log-transformed concentrations. LC values represent the lethal concentrations causing 30% and 50% mortality, respectively, with 95% confidence intervals (CI). The regression equation expresses the relationship between probit mortality (y) and $\log_{10}$-transformed concentration (x).

Table S2. Tukey HSD test results for reproductive performance in *Baryscapus dioryctriae*

Indicator	Group 1	Group 2	Mean Difference	Adjusted *p*-value	Significance
Parasitism rate	F0_LC50	F0_CK	-24.01	0.0022	**
	F0_LC50	F1_CK	-24.58	0.0016	**
Emergence rate	F0_LC50	F0_CK	-31.98	0.0003	***
	F0_LC50	F1_CK	-18.48	0.0321	*
Offspring per female	F0_LC50	F0_CK	-9.80	<0.0001	***
	F0_LC50	F0_LC30	-9.63	<0.0001	***
	F0_LC50	F1_CK	-8.55	0.0002	***
	F0_LC50	F1_LC30	-7.73	0.0004	***
	F1_LC50	F0_CK	-8.52	0.002	***
	F1_LC50	F0_LC30	-8.35	0.0003	***
	F1_LC50	F1_CK	-7.32	0.0005	***
	F1_LC50	F1_LC30	-6.50	0.0010	**

Note: Results are based on Tukey's Honest Significant Difference (HSD) test conducted across six treatment groups (F_0 CK, F_0 LC30, F_0 LC50, F_1 CK, F_1 LC30, F_1 LC50). Only statistically significant pairs shown ($p<0.05$). CK=Control, LC30=Sublethal exposure, LC50=High exposure. * = $p < 0.05$, ** = $p < 0.01$, *** = $p < 0.001$.

Table S3. Two-way ANOVA summary for reproductive performance of *Baryscapus dioryctriae*

Parameter	Factor	*F*	*p*-value	Significance
Parasitism rate	Treatment	8.942	0.0042	**
	Generation	27.641	< 0.0001	***
	Interaction	0.971	0.4051	ns
Emergence rate	Treatment	9.087	0.0039	**
	Generation	3.731	0.0763	ns
	Interaction	0.792	0.4715	ns
Female ratio	Treatment	1.827	0.1973	ns
	Generation	1.038	0.3272	ns
	Interaction	0.142	0.8687	ns
Offspring per female	Treatment	43.265	< 0.0001	***
	Generation	0.063	0.8057	ns
	Interaction	16.248	0.0003	***

Note: Two-way ANOVA was performed to examine the main effects of Treatment (CK, LC_{30}, LC_{50}), Generation (F_0 vs F_1), and their interaction on reproductive performance (n=3 per group). ns = not significant, * = $p < 0.05$, ** = $p < 0.01$, *** = $p < 0.001$. Degrees of freedom: Treatment (df=2,12), Generation (df=1,12), Interaction (df=2,12).

Table S4. Pairwise Tukey HSD test results for developmental durations of *Baryscapus dioryctriae*

Comparison	group1	group2	meandiff	*p*-adj	reject
Egg–larva duration	F0_CK	F0_LC30	-1.3333	0.3533	False
	F0_CK	F0_LC50	0.3333	0.9941	False
	F0_LC30	F0_LC50	1.6667	0.1677	False
	F1_CK	F1_LC30	0.0	1.0	False
	F1_CK	F1_LC50	-2.6667	0.0126	True
	F1_LC30	F1_LC50	-2.6667	0.0126	True
	F0_LC50	F1_LC50	-2.3333	0.0303	True[a]
Pupa–adult duration	F0_CK	F0_LC30	0.3333	0.4215	False
	F0_CK	F0_LC50	-1.6667	0.0	True
	F0_LC30	F0_LC50	1.5	0.0001	True
	F1_CK	F1_LC30	-0.3333	0.4215	False
	F1_CK	F1_LC50	-2.0000	<0.001	True
	F1_LC30	F1_LC50	-1.6667	<0.001	True

Note: '*p*-adj' shows adjusted *p*-values using Tukey's HSD correction. The 'reject' column indicates whether the null hypothesis of equal means is rejected at $\alpha = 0.05$. [a] Significant interaction is detected.

Table S5. Enzyme activities in *Baryscapus dioryctriae* adults under beta-cypermethrin exposure

Time (h)	GST (nmol/min/g FW)		AchE (nmol/min/g FW)		CarE (U/g FW)		CAT (μmol/min/g FW)		POD (U/g FW)		SOD (U/g FW)	
	Control	Treatment	Control	Treatment	Control	Treatment	Control	Treatment	Control	Treatment	Control	Treatment
24	607.68 ± 3.05	1349.56 ± 7.45	40.70 ± 1.50	32.12 ± 0.30	12.46 ± 0.25	20.75 ± 0.15	1212.61 ± 26.45	462.58 ± 7.38	3.30 ± 0.08	2.04 ± 0.03	3380.67 ± 32.15	2163.96 ± 18.43
48	628.34 ± 5.72	1040.04 ± 4.23	35.46 ± 1.45	38.97 ± 0.47	13.99 ± 0.25	16.65 ± 0.30	976.09 ± 38.84	713.92 ± 12.23	1.99 ± 0.03	1.58 ± 0.08	3916.96 ± 53.91	2361.66 ± 35.44
72	765.62 ± 13.77	1224.75 ± 7.69	41.07 ± 0.83	28.77 ± 0.36	13.59 ± 0.31	17.26 ± 0.09	1075.22 ± 35.28	545.94 ± 16.33	1.40 ± 0.03	2.52 ± 0.06	4132.40 ± 55.00	3798.31 ± 88.64
96	1420.05± 12.04	1177.72 ± 8.24	65.18 ± 1.63	50.92 ± 2.02	17.55 ± 0.18	17.30 ± 0.27	1142.06 ± 49.05	194.57 ± 4.89	3.63 ± 0.08	1.22 ± 0.04	3566.36 ± 97.45	2909.83 ± 54.22
120	795.93 ± 5.67	1237.11 ± 20.05	37.01 ± 0.73	44.51 ± 0.72	12.74 ± 0.27	15.49 ± 0.49	911.05 ± 39.90	601.20 ± 30.63	1.47 ± 0.05	0.94 ± 0.02	3265.60 ± 38.08	3092.48 ± 86.76
144	966.86 ± 5.10	1068.69 ± 42.69	51.80 ± 2.39	39.60 ± 0.71	18.01 ± 0.14	17.63 ± 0.26	1010.89 ± 28.75	353.35 ± 1.77	1.55 ± 0.05	1.00 ± 0.02	3385.81 ± 29.06	2983.14 ± 80.02
168	973.27 ± 13.83	1267.25 ± 19.90	29.32 ± 0.84	100.88 ± 1.66	17.13 ± 0.25	30.26 ± 1.33	520.00 ± 9.01	499.31 ± 2.29	2.72 ± 0.06	1.54 ± 0.04	3038.30 ± 32.72	2631.52 ± 50.79

Note: Enzyme activities are expressed as mean ± SE (n = 3). FW: fresh weight. Control: control group; Treatment: beta-cypermethrin treatment group (LC_{30}). GST: glutathione S-transferase; AchE: acetylcholinesterase; CarE: carboxylesterase; CAT: catalase; POD: peroxidase; SOD: superoxide dismutase.

Table S6. Two-way ANOVA summary for enzyme activities of *Baryscapus dioryctriae*

Enzyme	Effect	Df	*F* value	*p*-value
GST	Treatment	1	1393.22	< 0.0001
	Time	6	161.61	< 0.0001
	Treatment × Time	6	200.60	< 0.0001
AchE	Treatment	1	54.07	< 0.0001
	Time	6	168.15	< 0.0001
	Treatment × Time	6	284.05	< 0.0001
CarE	Treatment	1	332.33	< 0.0001
	Time	6	102.95	< 0.0001
	Treatment × Time	6	61.73	< 0.0001
CAT	Treatment	1	1235.50	< 0.0001
	Time	6	41.33	< 0.0001
	Treatment × Time	6	73.02	< 0.0001
POD	Treatment	1	607.87	< 0.0001
	Time	6	188.03	< 0.0001
	Treatment × Time	6	193.19	< 0.0001
SOD	Treatment	1	463.28	< 0.0001
	Time	6	86.05	< 0.0001
	Treatment × Time	6	38.13	< 0.0001

Note: "Treatment" refers to beta-cypermethrin exposure (LC_{30}); "Time" indicates sampling hours post-treatment (24–168 h); "Treatment × Time" refers to the interaction between treatment and time. All *p*-values < 0.0001 indicate statistically significant effects. DF for all effects: Treatment (df=1), Time (df=6), Interaction (df=6), Error (df=36).

Table S7. Levels of β-cypermethrin and its primary metabolite 3-phenoxybenzoic acid (PBA) in *Baryscapus dioryctriae* adults after LC_{30} exposure

Time (h)	PBA (live)	PBA (dead)	β-cypermethrin (live)	β-cypermethrin (dead)
24	51.73 ± 2.22	59.36 ± 1.58	26.01 ± 0.53	142.54 ± 3.04
48	45.13 ± 0.35	207.42 ± 4.64	–	–
72	238.72 ± 2.78	157.25 ± 5.57	24.41 ± 0.40	111.82 ± 2.15
96	106.43 ± 4.91	187.98 ± 2.86	–	–
120	127.52 ± 2.39	231.20 ± 9.83	20.02 ± 0.01	78.90 ± 3.64
144	151.31 ± 2.07	221.26 ± 13.49	–	–
168	30.62 ± 0.43	317.86 ± 9.44	32.43 ± 0.63	41.26 ± 0.44

Note: Values are presented as mean ± standard error (SE, n = 3). "Live" and "Dead" refer to parasitoid samples collected alive or deceased at each time point, respectively. "–" indicates unmeasured values.

Table S8. Summary of statistical test results (PBA vs β-cypermethrin)

Test type	Effect / Time (h)	PBA	β-cypermethrin
t-test (live *vs* dead)	24	0.048	<0.001
	48	<0.001	-
	72	<0.001	<0.001
	96	<0.001	-
	120	<0.001	<0.001
	144	<0.001	-
	168	<0.001	<0.001
Two-way ANOVA	Time main effect	0.0001	<0.0001
	Status main effect	<0.0001	<0.0001
	Time × Status	0.0022	0.0158

Note: *t*-tests were performed for each time point (live vs dead) using independent samples (equal variance if Levene's test passed; Welch's correction otherwise). Two-way ANOVA was performed with "time" and "status" as factors; *p*-values are shown for each effect. A dash (–) indicates that no value was provided in the original table.

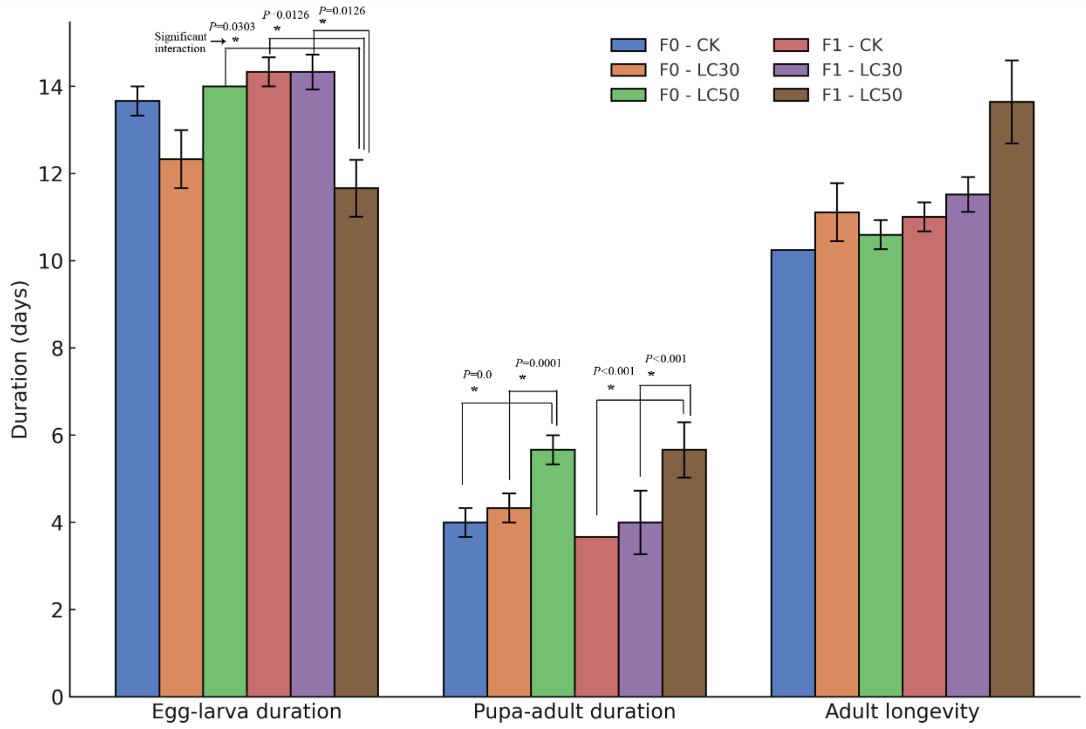

Figure S1. Effects of sublethal β-cypermethrin exposure on the developmental durations and adult longevity of *Baryscapus dioryctriae*. Bar plots show the egg–larva duration, pupa–adult duration, and adult longevity across two generations (F_0 and F_1) under control (CK), LC_{30}, and LC_{50} treatments. Data are presented as mean ± SE (n = 3 biological replicates). Significant differences between groups were determined using Tukey's HSD test ($p < 0.05$), with asterisks and p-values indicated above the corresponding bars (see Table S4 for detailed statistics).

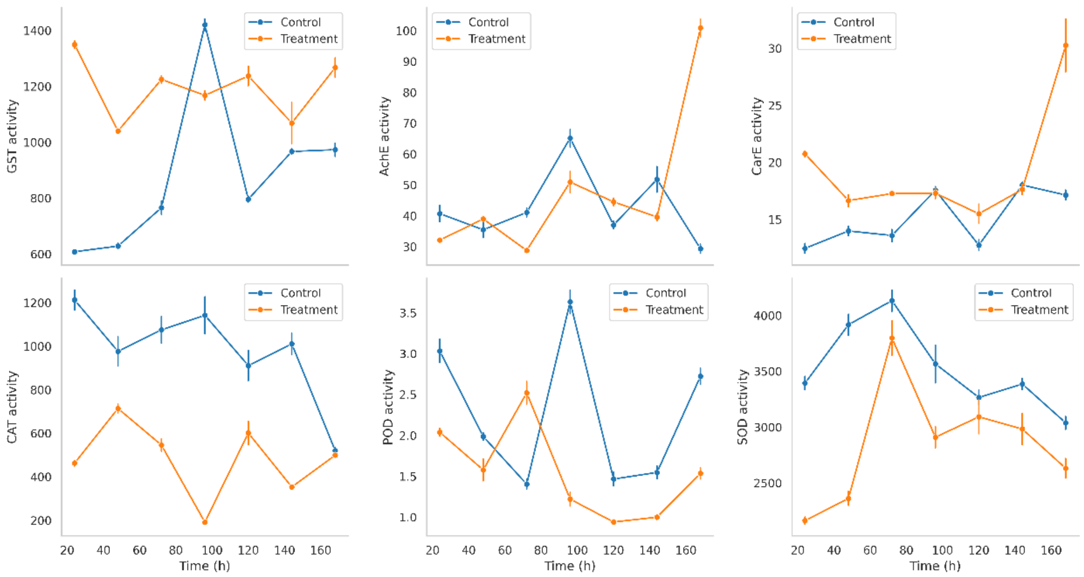

Figure S2. Trends in the activities of six key enzymes in *Baryscapus dioryctriae* adults under beta-cypermethrin (LC_{30}) exposure across seven time points (24–168 h). Each panel represents one enzyme: glutathione S-transferase (GST), acetylcholinesterase (AchE), carboxylesterase (CarE), catalase (CAT), peroxidase (POD), and superoxide dismutase (SOD). Data are presented as mean ± standard error (n = 3). Solid lines denote control and treatment groups. Significant interaction effects (treatment × time) were detected for all enzymes (Two-way ANOVA, $p < 0.0001$). Full enzyme activity values are provided in Table S5.
